# Thoracic disc herniation causing transient paraplegia coincident with epidural anesthesia: a case report

**DOI:** 10.4076/1757-1626-2-6228

**Published:** 2009-08-05

**Authors:** Arzu Kaya, Salih Ozgocmen

**Affiliations:** Department of Physical Medicine and Rehabilitation, Firat UniversityFaculty of Medicine, 23119 ElazigTurkey

## Abstract

Neurological deficits following epidural or spinal anesthesia are extremely rare. Transient paraplegia following epidural anesthesia in a patient with thoracic disc herniation has been presented. A 44-year-old woman developed paraplegia during the operation for vascular surgery of her legs under epidural anesthesia. Epidural hematoma or spinal cord ischemia was ruled out by magnetic resonance imaging of the thoracic and lumbar spine in which protruded disc at T11-12 level compressing the spinal cord has been verified. Patient responded well to steroid treatment and rehabilitation interventions. Physicians should be aware of preceding disc protrusions, which may have detrimental effects on spinal cord perfusion, as a cause of persistent or transient paraplegia before epidural anesthesia procedure. MRI is a valuable imaging option to rule out epidural anesthesia complications and coexisting pathologies like disc herniations.

## Introduction

Spinal or epidural anesthesia is a frequently preferred alternative to general anesthesia usually during orthopedic and peripheral (or minor) vascular surgery. Permanent neurological deficits following epidural or spinal anesthesia are extremely rare with an estimated incidence of 0.02% or less while transient neurological deficits are 0.1% [1-3]. The possible causes of these neurological deficits are miscellaneous including epidural hematoma or abscess formation, spinal infarcts or direct traumas, accidental neurotoxicity related to subaracnoid injections or chemical contamination [4].

Thoracic disc herniations are rare because of the special anatomical and biomechanical properties of the region [5,6]. The fact that diameter of the spinal canal is narrower, disc herniations at these levels - although rare - frequently cause myelopathy [5,7]. Herein we presented a case of transient paraplegia following epidural anesthesia for vascular surgery of the leg.

## Case presentation

**Figure 1.**
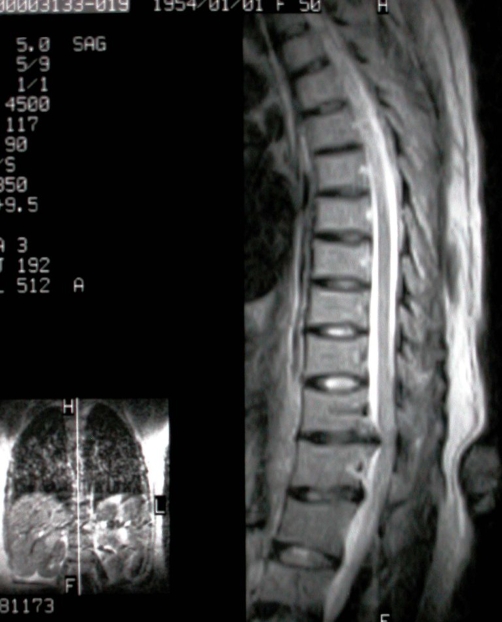
Magnetic resonance of the thoracic spine (T2-weighted, sagittal) shows disc protrusion at T11-12 level with osteophytes intruding the spinal canal.

**Figure 2.**
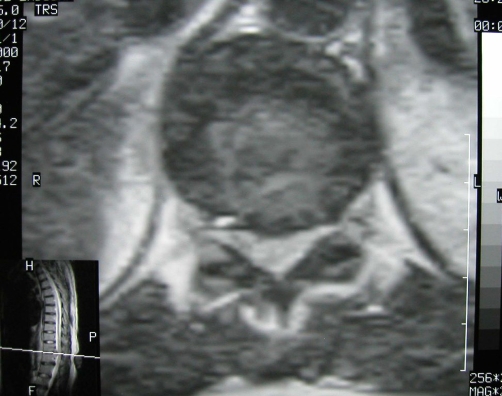
Magnetic resonance of the thoracic spine at T11-12 level (T2-weighted, axial) shows herniated nucleus pulposus and spinal cord compression.

A 44-year-old Turkish woman admitted to the department of vascular surgery for her leg varices. She was on treatment with pentoxifylline and etodolac and her coagulation status was normal (prothrombin time=11 s, partial thromboplastin time = 25 s). The patient placed on the table in sitting position and her back was prepared and draped in the operating room. The patient instructed for full cervical and lumbar flexion, lumbar 4-5 intervertebral space was infiltrated with 1% lidocaine and a midline epidural catheter was easily placed into the L4-5 interspace using *loss of resistance* technique. No blood or cerebrospinal fluid was aspirated from the epidural space. An anesthetic test dose 5 ml of 2% lidocaine was injected and then continued 18 ml of lidocaine 2%. An anesthetic level to T11 was obtained. No adverse reaction was noted. During the operation, the patient felt motor loss in her legs. Epidural anesthesia was stopped and the procedure was continued and completed under general anesthesia. During the 4-h course in post-anesthesia care unit and 6-h course in the neurology intensive care unit, the patient’s sensory block decreased from T11 to L1 level, but motor block persisted. There was symmetrical loss of reflexes and flaccid paraplegia in the legs. Lower extremity sensation to light touch and pain returned, and the patient complained of pain at the dorsolumbar region. The patient urgently underwent magnetic resonance imaging (MRI), with suspicious epidural hematoma or spinal cord compression or ischemia. Thoracic and lumbar MRI images revealed intervertebral disc protrusion at T11-T12 level and an outstanding spinal cord compression (Figure 1 and 2). There was no evidence of hematoma and the spinal cord intensity was normal throughout the thoracic spine which ruled out spinal cord ischemia. There were osteophytes of the vertebral endplates in different levels of thoracic and lumbar spine. The patient was given methylprednisolone and transferred to the rehabilitation unit. Her sphincter control and anal reflex was intact, and tonus of the anal sphincter was normal. Over ten days of rehabilitation including range of motion and strengthening exercises, electrotherapy to the dorsal region and superficial heat, the patient’s dorsolumbar pain reduced, motor recovery was achieved with a weakness of 3/5 of the lower extremities. The patient was discharged after 20 days of rehabilitation with minimal weakness of the lower extremities and independent in daily living activities.

## Discussion

Symptomatic thoracic disc herniation is an uncommon problem. Although the reported incidence of symptomatic thoracic disc herniations is relatively low, asymptomatic thoracic disc protrusions are estimated to be as high as 37% [8]. Protruded thoracic disc causing cord compression is estimated to 0.25-0.75 per cent of all symptomatic intervertebral discs [9]. Asymptomatic thoracic disc herniations are accidentally diagnosed with MRI.

Our case illustrates a demonstration to remember potential causes of transient or permanent neurological deficits following epidural anesthesia. Complications of regional spinal anesthesia are; intravascular injection of anesthetic media throughout spinal veins causing toxicity, inaccurately introduced anesthetic agent into the subarachnoid space which may cause central nervous system (CNS) dysfunction, high level anesthesia causing respiratory or CNS dysfunction, and dorsal pain or post-anesthesia headache. Potential causes of paraplegia coincident with spinal anesthesia includes: epidural and subdural hematoma associated with systemic heparinization or platelet dysfunctions, spinal cord ischemia associated with thrombosis of anterior spinal artery, spinal cord traumas related to direct needle injuries or chemical toxicity of the anesthetic agents [10-14,15]. In our case, lack of evidence for hematoma and normal signal intensity of the spinal cord in MRI ruled out these complications. In a recent report by Chan et al an elderly male in whom hypotension and sudden onset paraplegia had occurred following epidural anesthesia has been presented. The patient has been reported to have atherosclerosis and thrombogenic risk factors that had an impact on spinal cord circulation. On repeated MRI scans diffuse ischemic findings have been demonstrated which were highly specific for spinal cord ischemia [12].

Blood supply of the spinal cord may be affected by combination of various factors [2,16]. There is a minimum perfusion pressure to maintain spinal perfusion and to prevent spinal cord infarction. Hypotension during epidural anesthesia is an expected complication but dropped systolic pressure more than 60-80 mmHg increases the risk of spinal cord ischemia. As in our case with hyperkyphosis during the procedure may increase the risk of spinal cord ischemia if there is an underlying precipitating factor like thoracic disc protrusion compressing the spinal cord. Besides, introducing excessive amount of anesthetics into the epidural space may cause an increase in cerebrospinal fluid pressure and vascular stasis as well [15]. Using adrenalin boost the effect of anesthetic’s activity but doses more than 1:160 000 dilution may cause thrombosis or vasospasm in the spinal cord vasculature [2,3,16]. Additionally, individual characteristics of the spinal cord vasculature and segmental deficiency of the anterior spinal artery may further precipitate the risk of ischemia in some patients [1].

Reactivation of earlier disc diseases with spinal procedures is reported to be rare [17,18]. In our patient, a large disc protrusion far from the catheter level without any additional pathology has been demonstrated on MRI. Based on medical records and patients self report, we found that patient’s thoracic disc herniation was asymptomatic prior to the operation. However, it is not clear that positioning for epidural procedure or surgical intervention had a role to aggravate disc protrusion or not.

In summary, we underscore the necessity to be aware of preceding disc protrusions or other factors, which had detrimental role in spinal cord perfusion, as a cause of persistent or transient paraplegia before epidural anesthesia.

## Abbreviations

CNS: cenral nervous system
MRI: magnetic resonance imaging
